# Preparation of Two Process-Related Impurities of a Key Intermediate of Silodosin Under Baeyer–Villiger and Fenton Conditions

**DOI:** 10.3390/molecules31030462

**Published:** 2026-01-28

**Authors:** Wenbin Chen, Qiang Zhou, Junjun Zhang, Jianyang Jin, Juan Zhang, Jiangbo Xi, Zhengwu Bai, Min Li

**Affiliations:** 1 School of Chemistry and Environmental Engineering, Wuhan Institute of Technology, Wuhan 430073, China; 20081703@wit.edu.cn (J.Z.); zhang_juan@wit.edu.cn (J.Z.); jbxi@wit.edu.cn (J.X.); 2 Center of Excellence for Modern Analytical Technologies (CEMAT), Zhejiang Huahai Pharmaceutical Co., Ltd., Linhai 317204, China; zhouqiang@huahaipharm.com (Q.Z.); jinjianyang@huahaipharm.com (J.J.); 3 Huahai US, Inc., 700 Atrium Drive, Somerset, NJ 08873, USA

**Keywords:** silodosin intermediate, process-related impurities, oxidation, Baeyer–Villiger oxidation, Fenton reaction

## Abstract

Control of process-related impurities is of critical importance for developing an efficient and suitable synthetic process of an active pharmaceutical ingredient. In the study of a key intermediate of silodosin (KIS), two process-related impurities including the benzaldehyde impurity (BAI) and indole impurity (IDI) were prepared and fully characterized to determine their downstream fate. Under optimized conditions, BAI was formed in a yield of ~48% by treating KIS with 10% hydrogen peroxide at 60 °C. Interestingly, BAI would not be expected to be the major product under the apparent Baeyer–Villiger oxidative condition. Furthermore, by adding 20 mM FeCl_3_ into the above 10% hydrogen peroxide solution, IDI became the major product in a yield of ~43% under this Fenton reaction-like condition. The probable formation mechanism of IDI was discussed and validated in the context of certain structurally similar substrates.

## 1. Introduction

The quality of pharmaceutical products directly impacts their efficacy and safety, with impurity profile being a critical quality attribute [1,2,3,4,5]. To develop an efficient and suitable synthetic process of an active pharmaceutical ingredient (API), the control of process-related impurities is of critical importance. Hence, it becomes necessary to understand the origins, downstream flow, and final purge of the process-related impurities at each key intermediate and all the way towards the final API.

**Figure 1:**
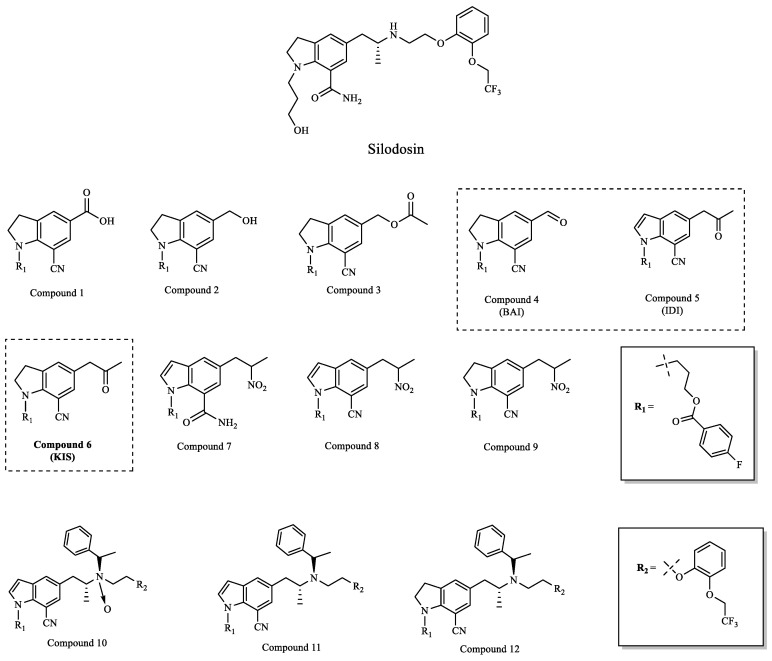
Structures of silodosin and compounds **1**–**12**. Compound 6 is the key intermediate of silodosin (KIS), while compounds 4 and 5 are the benzaldehyde impurity (BAI) and indole impurity (IDI).

Silodosin is an α-adrenergic receptor antagonist and primarily acts on the α1A adrenergic receptors [6,7]. It is used clinically to treat symptoms associated with benign prostatic hyperplasia (BPH). It is chemically known as 1-(3-hydroxypropyl)- 5-[(2*R*)-2-[2-[2-(2,2,2-trifluoroethoxy)phenoxy]ethylamino]propyl]-2,3-dihydro-1*H*-indole-7-carboxamide (Figure 1).

In order to formulate an effective control strategy, the preparation of two process-related impurities for the key intermediate of silodosin (KIS), the benzaldehyde impurity (BAI) and indole impurity (IDI), was a crucial requirement to understand their formation mechanisms, and to determine their downstream fate [8,9,10]. For the latter purpose, the two impurities were required as reference materials to develop and validate the analytical methods to quantitatively determine their downstream flow and purge [11,12]. Nevertheless, the two compounds have never been reported in the literature.

In the early phase of developing analytical methods for KIS, it was subjected to a forced degradation study, including the oxidative stress with 10% hydrogen peroxide under ambient conditions, in which both BAI and IDI were formed in very low yields. By following the initial lead, much-improved preparative conditions were obtained for BAI and IDI, respectively. In this paper, we not only describe the preparation and full characterization of BAI and IDI, but also discuss their probable formation mechanisms. The results were somewhat surprising because BAI was not expected to be the major product under the apparent Baeyer–Villiger oxidation condition [13,14,15,16]. On the other hand, IDI was produced as the major product under a Fenton reaction-like condition. The validity of the proposed formation mechanism for IDI is discussed in the context of certain structurally similar substrates.

## 2. Results and Discussion

### 2.1. LC-PDA/UV-High-Resolution MS^n^ (n = 1, 2) Analysis of the Key Intermediate of Silodosin (KIS)

**Figure 2:**
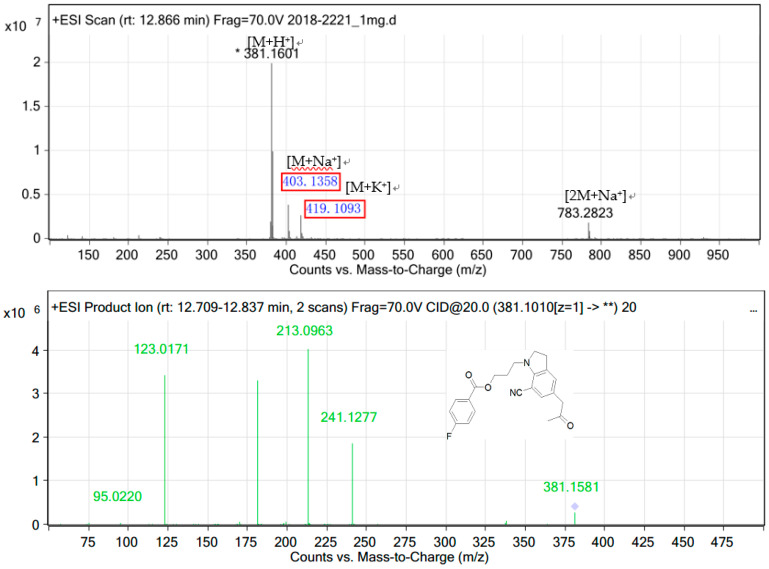
MS^1^ (**upper**) and MS^2^ (**lower**) spectra of KIS.

**Scheme 1:**
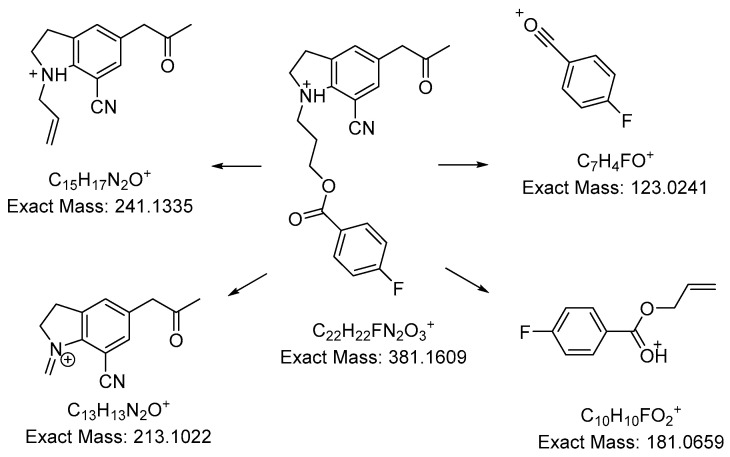
Proposed pathways for the MS^2^ fragmentation of KIS.

In ESI positive mode, the mass spectrometry signal for KIS showed a precursor ion at *m*/*z* 381.1601, corresponding to the molecular formula of C_22_H_21_FN_2_O_3_ with an error of 2 ppm (Figure 2). The peaks at *m*/*z* 403 and 419 correspond to the [M+Na^+^] and [M+K^+^] adducts of KIS, respectively, while the signal at *m*/*z* 783 is assigned to the [2M+Na^+^] ion of KIS, a gas phase dimeric ion. MS^2^ analysis revealed four primary product ions with *m*/*z* values of 241.1277, 213.0963, 181.0594, and 123.0171, respectively. The fragment *m*/*z* 241.1277 (C_15_H_17_N_2_O^+^) results from the loss of one molecule of *p*-fluorobenzoic acid from KIS. The ion at *m*/*z* 213.0963 (C_13_H_13_N_2_O^+^) differs from *m*/*z* 241 by the loss of an ethyl group. The ion at *m*/*z* 181.0594 (C_10_H_10_FO_2_^+^) corresponds to propyl *p*-fluorobenzoic acid, and the ion at *m*/*z* 123.0171 (C_7_H_4_FO^+^) is associated with *p*-fluorobenzaldehyde. Based on the observed fragmentation patterns and high-resolution mass data, the proposed fragmentation pathways of KIS are shown in Scheme 1.

### 2.2. LC-PDA/UV-MS^n^ (n = 1, 2) Analysis of Benzaldehyde Impurity (BAI)

**Figure 3:**
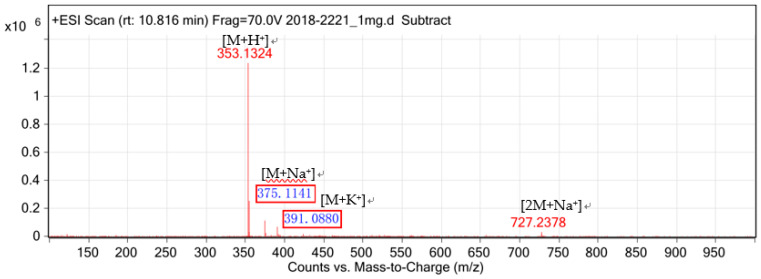

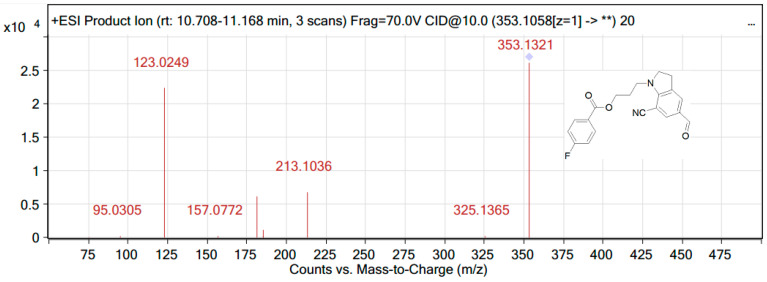
MS^1^ (**upper**) and MS^2^ (**lower**) spectra of BAI.

**Scheme 2:**
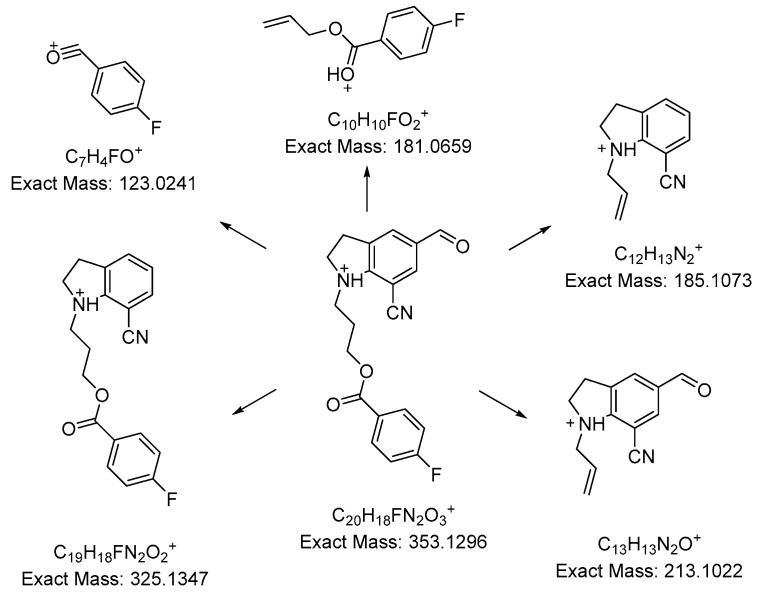
Proposed pathways for the MS^2^ fragmentation of BAI.

The MS spectrum of BAI revealed a [M+H^+^] mass signal at *m*/*z* 353.1324, corresponding to a molecular formula of C_20_H_17_FN_2_O_3_ with an error of 8 ppm (Figure 3). The signals at *m*/*z* 375 and 391 correspond to the [M+Na^+^] and [M+K^+^] adducts of BAI, respectively, while the signal at *m*/*z* 727 is assigned to the [2M+Na^+^] ion of BAI. This molecular formula is consistent with the loss of a C_2_H_4_ unit relative to that of KIS (C_22_H_21_FN_2_O_3_). The main MS^2^ product ions observed were *m*/*z* 325.1365 (C_19_H_18_FN_2_O_2_^+^), *m*/*z* 213.1036 (C_13_H_13_N_2_O^+^), *m*/*z* 185.1066 (C_12_H_13_N_2_^+^), *m*/*z* 181.0672 (C_10_H_10_FO_2_^+^), and *m*/*z* 123.0249 (C_7_H_4_FO^+^). Notably, the fragments at *m*/*z* 123 and *m*/*z* 181 were also found in KIS, suggesting that the impurity structure likely contained a *p*-fluorophenyl acetate moiety. The fragment at *m*/*z* 325 results from the loss of CO from the precursor ion, while the fragments at *m*/*z* 213 and *m*/*z* 185 differ by the loss of CO, indicating the possible presence of aldehyde groups within the BAI structure. Furthermore, the *m*/*z* 185 fragment results from the loss of C_8_H_5_FO_3_, which corresponds to one molecule of *p*-fluorobenzoic acid (C_7_H_5_FO_2_) and one molecule of formaldehyde (CH_2_O). The fragmentation pathways for BAI are proposed in Scheme 2.

### 2.3. LC-PDA/UV-High-Resolution MS^n^ (n = 1, 2) Analysis of the Indole Impurity (IDI)

**Figure 4:**
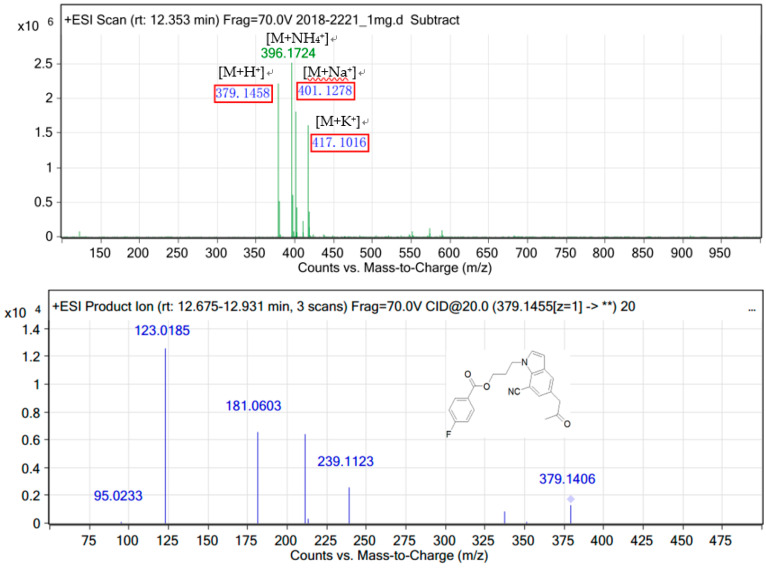
MS^1^ (**upper**) and MS^2^ (**lower**) spectra of IDI.

**Scheme 3:**
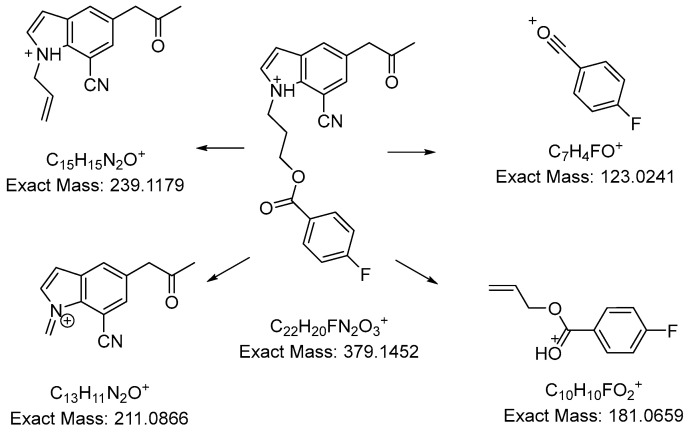
Proposed pathways for the MS^2^ fragmentation of IDI.

A strong ion peak at *m*/*z* 379.1458 was observed, corresponding to the [M+H] peak of IDI, matching a molecular formula of C_22_H_19_FN_2_O_3_ within an error of 2 ppm (Figure 4). The peaks at *m*/*z* 396, 401, and 417 correspond to the [M+NH_4_^+^], [M+Na^+^], and [M+K^+^] adducts of IDI, respectively. Compared to the molecular formula of KIS (C_22_H_21_FN_2_O_3_), IDI exhibits a loss of two hydrogens (2H). The primary product ions detected were *m*/*z* 239.1123 (C_19_H_18_FN_2_O_2_^+^), *m*/*z* 211.0807 (C_13_H_11_N_2_O^+^), *m*/*z* 181.0603 (C_10_H_10_FO_2_^+^), and *m*/*z* 123.0185 (C_7_H_4_FO^+^). Additionally, the product ions at *m*/*z* 123 and *m*/*z* 181 are consistent with the MS/MS fragments of KIS, suggesting that the structure of IDI contains a propyl *p*-fluorobenzoate moiety. Furthermore, it is noteworthy that the fragments at *m*/*z* 211 and *m*/*z* 239 for IDI differ by exactly two hydrogen atoms (2H) from those at *m*/*z* 213 and *m*/*z* 241 associated with KIS. The fragmentation pathways for IDI are proposed in Scheme 3.

### 2.4. Optimization for Formation of the Benzaldehyde Impurity (BAI) and Indole Impurity (IDI)

**Figure 5:**
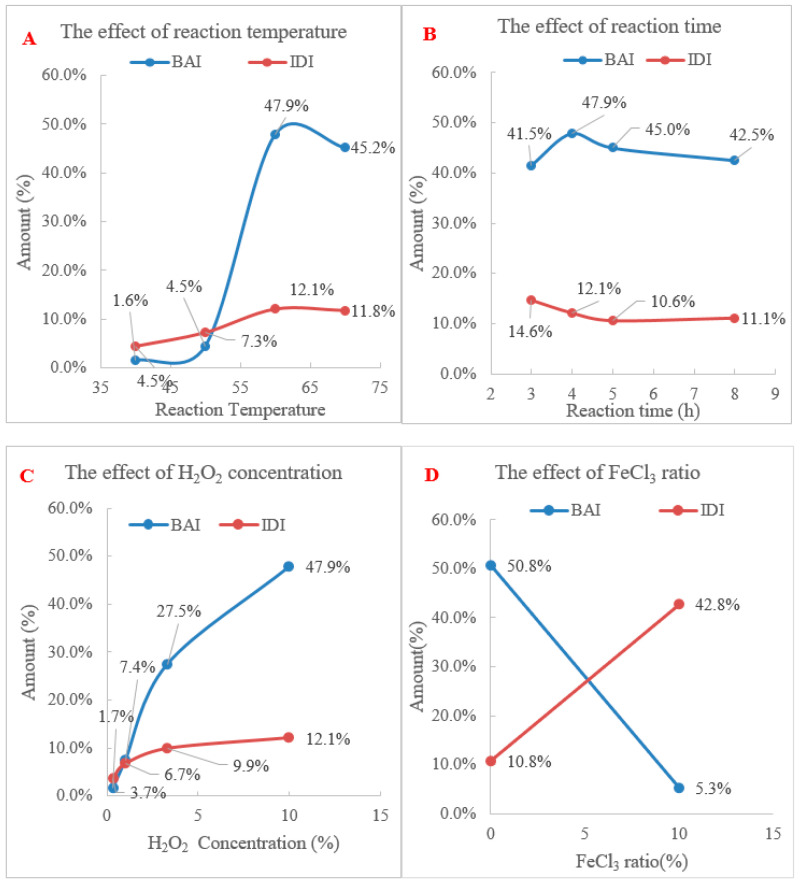
Effect of different conditions on the formation of the benzaldehyde impurity (BAI) and indole impurity (IDI) from the key intermediate of silodosin (KIS): (**A**) reaction temperature, (**B**) reaction time, (**C**) H_2_O_2_ concentration, and (**D**) in the presence of FeCl_3_.

This optimization study systematically examined the formation of the two key oxidative impurities of KIS, benzaldehyde impurity (BAI) and indole impurity (IDI), under various reaction conditions. The results reveal distinct pathways and condition dependencies for their generation. The detailed data can be found in Supporting Materials (Figures S1) and Figure 5A–D. Briefly, BAI formation was predominantly driven by elevated temperature (40–60 °C) and H_2_O_2_ concentration (0.33–10%), with yields increasing sharply from 1.6% to ~48% and 1.7% to ~48%, respectively, indicating strong thermal and oxidative dependence. When FeCl_3_ was used as the sole oxidant, BAI yield reached only 2.3%, underscoring the limited contribution of iron-mediated oxidation in the absence of H_2_O_2_ (Supporting Materials, Figure S2). A reaction duration of 4 h was determined to be optimal; extending the reaction time beyond this point resulted in a slight decrease in the yields of both BAI and IDI.

In contrast, IDI formation critically depended on the synergistic action of FeCl_3_ and H_2_O_2_, characteristic of a Fenton-like mechanism. It was not observed as a major product in the absence of either component. IDI formation was moderately raised from 4.5% to 12.1% in the temperature range of 40–60 °C. Under optimized conditions (20 mM FeCl_3_, 10% H_2_O_2_, 60 °C, 4 h), IDI yield reached ~43%, significantly exceeding the ~12% yield obtained with a single oxidant condition (Supporting Materials, Figure S2). This pronounced difference highlights the essential role of Fe^3+^ in directing reaction selectivity toward IDI formation.

Consequently, the optimal synthesis conditions for BAI were determined to be 60 °C, 10% H_2_O_2_ concentration, and a reaction duration of 4 h. For IDI synthesis, the optimal conditions were established as a reaction temperature of 60 °C, a duration of 4 h, and the use of 20 mM FeCl_3_ and 10% H_2_O_2_.

### 2.5. Preparation and Isolation of BAI and IDI

Based on the findings of the optimization study, a larger-scale preparation of BAI was carried out as follows: First, 1.0 g of the key intermediate of silodosin (KIS) was added to a 100 mL round-bottom flask, followed by 10 mL of a 30% H_2_O_2_ aqueous solution and 20 mL of acetonitrile. The mixture was shaken in a dry heater at 60 °C for 4 h. The reaction mixture was extracted with ethyl acetate and the organic phase was evaporated to dryness, yielding a black powder, which was purified by silica gel column chromatography using a mixed solvent system of petroleum ether and ethyl acetate with a volume ratio of 3:1. The fractions containing the target product were combined and evaporated to yield a solid of ~300 mg with an overall isolated yield of ~33%.

A larger-scale preparation of IDI was carried out as follows: First 1.0 g of the key intermediate of silodosin (KIS) was added into a reaction flask, followed by addition of 10 mL of 30% H_2_O_2_ aqueous solution and 10 mL of FeCl_3_ solution (10 mg/mL in 80% acetonitrile aqueous solution) and 10 mL of acetonitrile. The mixture was shaken in a dry heater at 60 °C for 4 h, followed by extraction with 15 mL of ethyl acetate twice. The organic phase was combined and back washed with 15 mL of water, and then evaporated in vacuo. The residue was loaded onto a silica gel column and eluted using a mixed solvent system consisting of petroleum ether and ethyl acetate with a volume ratio of 3:1. The target product was obtained in a quantity of 340 mg with an overall isolated yield of ~34%.

### 2.6. One-Dimenional and Two-Dimensional NMR Characterization of BAI and IDI

**Figure 6:**
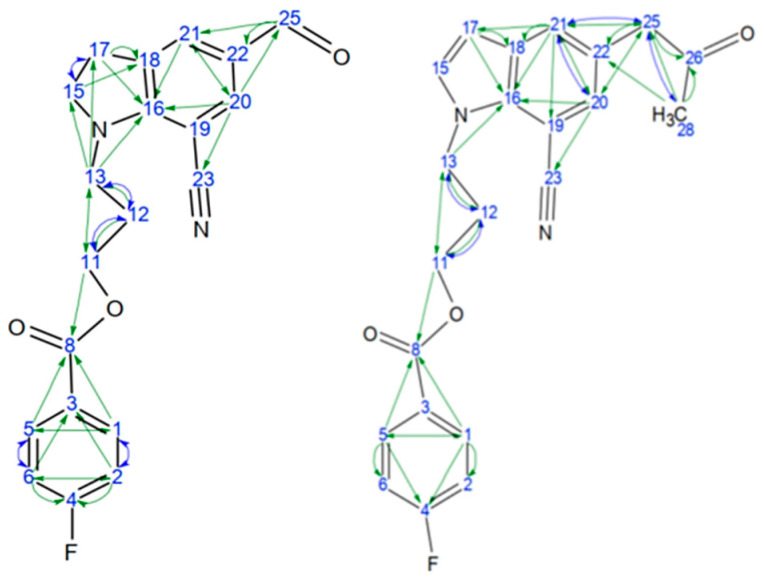
COSY and HMBC correlation NMR spectra for BAI and IDI are presented, respectively (Blue: COSY, Green: HMBC).

Figure 6 and Table 1 present the two-dimensional NMR correlation spectra and ^1^H and ^13^C signal assignments for BAI and IDI, respectively (Supporting Materials, Figures S5–S7).

Compared with the NMR spectrum of KIS, the absence of signals for C-26 and C-28 in BAI, together with the downfield resonance of C-25 at δ 188.9 ppm, provides clear evidence of a pronounced deshielding effect, consistent with the formation of a carbonyl functional group. Moreover, the close similarity observed in the remaining NMR signals between IDI and KIS indicates that the core molecular framework remains essentially unchanged, further supporting the conclusion that structural modification is confined to the site of oxidation. This distinct spectroscopic pattern is in good agreement with the molecular structure deduced from mass spectrometric analysis, thus providing robust support for the structural assignment of BAI.

In IDI, the pronounced downfield shifts in H-15 (δ 7.57 ppm) and H-17 (δ 6.587 ppm) relative to their counterparts in KIS are consistent with aromatization induced by oxidation of the dihydroindole ring. The high degree of similarity in the remaining NMR signals between IDI and KIS further corroborates that IDI arises specifically from oxidative transformation of the dihydroindole moiety in KIS, without extensive alteration to the rest of the molecular framework.

### 2.7. Probable Formation Mechanism of BAI

**Scheme 4:**
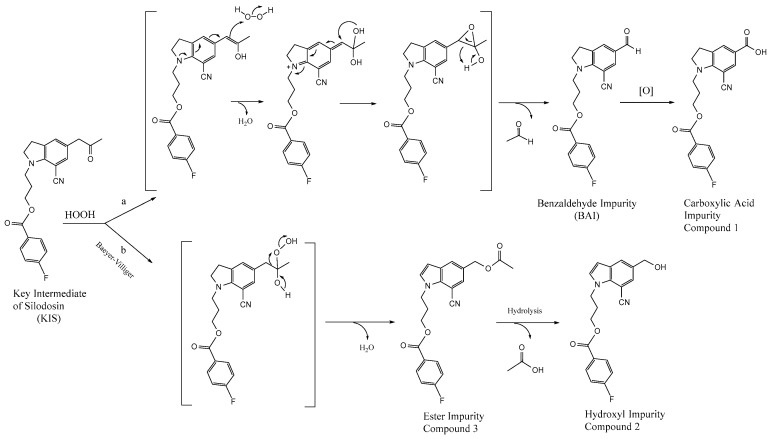
Plausible oxidation pathways of the key intermediate of silodosin (KIS) in hydrogen peroxide solutions.

Under the forced degradation conditions that predominantly yield BAI, Baeyer–Villiger oxidation is theoretically expected to occur. However, these results were somewhat surprising, because BAI would not be expected to be the major product under this apparent Baeyer–Villiger oxidative condition, except that no acid (including Lewis acid) was used as a catalyst [6,7]. In a Baeyer–Villiger oxidation by hydrogen peroxide such as the case in the present study, KIS would be expected to form the corresponding ester (Entry 3 in Table 2) and related compounds as the major products (Scheme 4). Although the ester and its subsequent hydrolytic degradant (Entry 2 in Table 2) were indeed formed (up to ~7% and ~2%, respectively), the major products were BAI (~48%) and its further oxidized degradant, the carboxylic acid impurity (Entry 1 in Table 2). Hence, the total yield of the products (the ester and carboxylic acid impurities) from the unexpected, non-Baeyer–Villiger oxidation was ~69%. It seems that the major driving force under the current reaction conditions would be an enamine-like nucleophilic oxidation towards hydrogen peroxide (Pathway a, Scheme 4), rather than hydrogen peroxide attacking the ketone group of KIS (Pathway b, Scheme 4), the latter of which would be expected in a typical Baeyer–Villiger oxidation. In order for the nucleophilic oxidation to take place, the ketone group of KIS would undergo an enolization process to produce the enol form, which should be facilitated by the resulting conjugation. The nucleophilic attack towards hydrogen peroxide by the newly formed enol double bond should be driven by the enamine functionality embedded in the dihydroindole moiety, leading to the formation of the diol, which would cyclize to produce the epoxide intermediate. Cleavage of the latter intermediate should lead to the formation of BAI (~48% yield), with simultaneous elimination of acetaldehyde, and apparently its further oxidative product, the carboxylic acid impurity (~21% yield). The distinct and reproducible distribution profile of these reaction products offers compelling experimental evidence in support of the Pathway a mechanism, thereby reinforcing its mechanistic coherence and scientific plausibility.

### 2.8. Probable Formation Mechanism of IDI

**Scheme 5:**
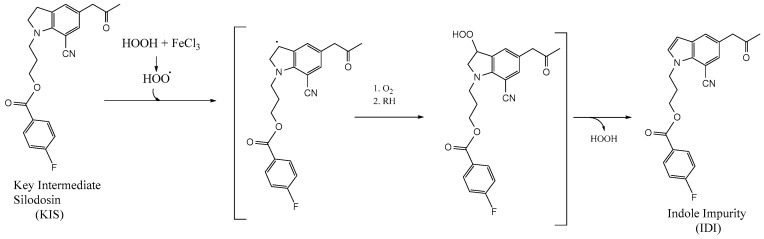
Plausible oxidation pathway of the key intermediate of silodosin (KIS) under a Fenton reaction-like condition (hydrogen peroxide solution with FeCl_3_).

With regard to the other process impurity IDI, among the attempts to improve its yield, 20 mM ferric chloride (FeCl_3_) was added into the 10% hydrogen peroxide solution at 60 °C for 4 h, under which condition IDI became the major product in 43% yield (Table 3) under this Fenton reaction-like condition. Since the discovery of the reaction in the end of the 19th century by Henry J. H. Fenton, the mechanism of this seemingly simple reaction has not been fully elucidated even as of today [17,18,19]. Nevertheless, it is generally agreed that in the presence of ferric chloride, H_2_O_2_ can undergo decomposition via a redox process, generating the peroxyl radical as the reactive intermediate (Scheme 5). The peroxyl radical would subsequently abstract a hydrogen from the benzyl position of the dihydroindole moiety. The resulting benzyl radical could react with molecular oxygen to form the peroxide intermediate, which would subsequently decompose into IDI via elimination of hydrogen peroxide.

It is worth to note that under this Fenton reaction-like condition, the competing minor pathways lead to the formation of BAI and the ester impurity remained, as evidenced by the formation of BAI (~5%) and its further oxidative degradant (**1**), the carboxylic impurity (~6%), and the formation of the ester impurity (**3**, ~3%) and its hydrolytic degradant, the hydroxyl impurity (**2**, ~1%).

To test the validity of the proposed mechanism for IDI formation, analogous compounds containing the same dihydroindole moiety (**9** and **12**, Figure 1) were employed as the substrates in the Fenton reaction-like oxidation. These two compounds were also the intermediates in the silodosin synthetic process. A solution of FeCl_3_ was added into the 10% hydrogen peroxide solutions containing **9** and **12**, respectively, and the resulting solutions, each containing 20 mM of FeCl_3_, were heated at 60 °C for 4 h. As illustrated in Table 4, compound **9** yielded ~36% of the corresponding indole degradant (**8**), while further hydrolysis of the cyano group of **8** gave **7** in ~17% yield. Collectively, the products containing the desired indole ring were formed in a total yield of ~53%. In the meantime, ~20% of the starting material (**9**) remained unchanged. On the hand, compound **12** generated only ~5% of the corresponding indole degradant (**11**, Table 5), and the primary product obtained was the *N*-oxide (**10**, Table 5, ~38%) due to further oxidation of **11** on its tertiary amine group. It is well-known that tertiary amines are quite susceptible to oxidation by hydrogen peroxide to afford *N*-oxides [17]. All the data are presented in Table 4 and Table 5, respectively.

## 3. Materials and Methods

### 3.1. Materials

The key intermediate of silodosin (KIS) and its two other intermediates (compounds **9** and **12**) were manufactured by Zhejiang Huahai Pharmaceutical Co., Ltd. (Linhai, China). HPLC-grade acetonitrile was purchased from Sigma-Aldrich Corp. (St. Louis, MO, USA). Hydrogen peroxide (30%) was purchased from Sinopharm Co., Ltd. (Beijing, China). Anhydrous ferric chloride was obtained from Aladdin Industrial Corporation (Shanghai, China), while trifluoroacetic acid (TFA) was supplied by ROE Scientific Inc. (Newark, NJ, USA).

### 3.2. Instrument Parameters

HPLC separation was carried out on a Thermo Ultimate 3000 HPLC system (Chromeleon 7.3.2 software) equipped with an Agilent SB-C18 column (150 × 4.6 mm, 5 μm) using a mobile phase system consisting of A (0.1% trifluoroacetic acid in H_2_O) and B (acetonitrile) (Agilent, Santa Clara, CA, USA). The analyses were performed at 30 °C with a flow rate of 1.0 mL/min and a gradient program varied according to the following program: 0 min (40% B), 15 min (40% B), 20 min (95% B), 22 min (95% B), 22.5 min (40% B), and 30 min (40% B). UV spectra were collected using a PDA/UV detector within a range spanning from 200 to 400 nm and UV chromatograms were collected at 225 nm.

### 3.3. LC-PAD/UV-Q TOF MS^n^ (n = 1 and 2) Experiments

High-resolution LC-PDA/UV-MS and -MS^2^ analyses were performed on an Agilent 6545 Q-TOF (MassHunter 7.0 software) mass spectrometer interfaced to an Agilent 1260 HPLC system equipped with a PDA/UV detector. The chromatographic conditions of the LC-PDA/UV-MS^n^ (n = 1, 2) methods were the same as those of the HPLC method.

The Q TOF mass spectrometer was operated in positive ESI mode under the following source parameters: fragmentor voltage at 70 V, drying gas flow 10 L/min and temperature maintained at 325 °C, nebulizer pressure set to 60 psi, sheath gas flow 12 L/min and sheath gas temperature at 350 °C, and capillary voltage fixed at 3.5 kV. The mass acquisition range was established from *m*/*z* 100 to 1700; for the MS^2^ analyses, the collision energy was set at levels of 10 eV, 20 eV, and 30 eV, respectively.

All the nuclear magnetic resonance data were acquired on an Agilent 400 MHz NMR instrument. Approximately 20 mg of the isolated degradant was dissolved in 1 mL of DMSO-*_d6_* and analyzed at 25 °C for all the NMR experiments. Standard Agilent pulse sequences (^1^H NMR, ^13^C NMR, gCOSY, gHSQC, and gHMBC.) were used to acquire the 1D and 2D NMR data (2D results presented in Supporting Materials).

## 4. Conclusions

The two process-related impurities for a key intermediate of silodosin have been synthesized and fully characterized as reference materials through simple and straightforward preparative procedures, enabling the development and validation of the analytical methods for the determination of their downstream fate. During the study, the probable mechanisms for the formation of the two process impurities were proposed and related degradants were examined, which are consistent with the proposed mechanisms. Based on the proposed mechanisms, it reveals that the enamine-driven nucleophilic oxidation via hydrogen peroxide may be applicable to the oxidation of structurally similar compounds, while the Fenton reaction-like oxidative condition has been demonstrated to be capable of oxidizing the dihydroindole moiety into an indole ring.

Based on the research presented in this paper, impurity reference standards were successfully obtained to support the development and validation of analytical methods. By elucidating the structures and formation mechanisms of two key process-related by-products and integrating these insights into the KIS synthesis pathway, process engineers have optimized the oxidation step through reduced reagent concentrations and lower reaction temperatures. This strategic modification has significantly minimized by-product formation, resulting in a measurable improvement in the overall yield of KIS.

## Figures and Tables

**Table 1:** NMR data of BAI and IDI.

Position	KIS		BAI		IDI	
	C Shift	H Shift	C Shift	H Shift	C Shift	H Shift
1, 5	132.2	8.07	132.3	8.00	132.2	7.91
2, 6	115.5	7.09	115.8	7.29	115.6	7.28
3	126.2	N.A	126.3	N.A	126.0	N.A
4	165.6	N.A	165.3	N.A	165.0	N.A
8	165.5	N.A	165.1	N.A	164.7	N.A
11	62.5	4.45	62.9	4.38	62.4	4.29
12	26.9	2.14	26.4	2.12	29.8	2.28
13	45.0	3.76	44.1	3.84	44.0	4.64
15	53.1	3.60	52.9	3.81	132.1	7.57
16	152.0	N.A	155.7	N.A	132.2	NA
17	27.1	2.97	25.7	3.01	102.0	6.58
18	132.9	N.A	134.4	N.A	130.3	N.A
19	87.8	N.A	85.2	N.A	92.3	N.A
20	131.8	6.94	140.2	7.79	129.6	7.45
21	129.5	6.93	125.0	7.46	127.6	7.70
22	122.5	N.A	126.2	N.A	126.0	N.A
23	119.1	N.A	118.4	N.A	118.3	N.A
25	49.2	3.53	188.9	9.55	48.4	3.87
26	206.2	N.A	N.A	N.A	206.1	N.A
28	29.3	2.16	N.A	N.A	29.5	2.15

Notes: N.A. stands for “Not Detected”.

**Table 2:** Product distribution of KIS in 10% H_2_O_2_ solution at 60 °C for 4 h.

Compound No.	Retention Time (min)	HPLC Result (%)	*m*/*z*	Formula
**1**	8.7	20.76	369.1	C_20_H_18_FN_2_O_4_^+^
**2**	9.7	2.23	355.1	C_20_H_20_FN_2_O_3_^+^
**3**	10.0	7.12	397.1	C_22_H_22_FN_2_O_4_^+^
**4**	11.2	47.92	353.1	C_20_H_18_FN_2_O_3_^+^
**5**	12.7	12.07	379.1	C_22_H_20_FN_2_O_3_^+^
**6**	13.1	5.92	381.1	C_22_H_22_F N_2_O_3_^+^

Notes: Compound **6** is the key intermediate of silodosin (KIS), compound **4** is the benzaldehyde impurity (BAI), and compound **5** is the indole impurity of KIS, i.e., IDI. The *m*/*z* values and formulae displayed are the protonated ions of the corresponding compounds.

**Table 3:** Product distribution of KIS in 10% H_2_O_2_ solution spiked with 20 mM FeCl_3_ at 60 °C for 4 h.

Compound No.	Retention Time (min)	HPLC Result (%)	*m*/*z*	Formula
**1**	8.2	6.10	369.1	C_20_H_18_FN_2_O_4_^+^
**2**	9.7	0.64	355.1	C_20_H_20_FN_2_O_3_^+^
**3**	10.2	3.42	397.1	C_22_H_22_FN_2_O_4_^+^
**4**	11.2	5.28	353.1	C_20_H_18_FN_2_O_3_^+^
**5**	12.7	42.82	379.1	C_22_H_20_FN_2_O_3_^+^
**6**	13.1	28.85	381.1	C_22_H_22_FN_2_O_3_^+^

Notes: The numbering of the compounds is continuous and unique throughout this paper. Compound **6** is the key intermediate of silodosin (KIS), compound **4** is the benzaldehyde impurity (BAI), and compound **5** is the indole impurity of KIS, i.e., IDI. The *m*/*z* values and formulae displayed are the protonated ions of the corresponding compounds.

**Table 4:** Product distribution of compound **9** subjected to 10% H_2_O_2_ solution spiked with 20 mM FeCl_3_ at 60 °C for 4 h.

Compound No.	Retention Time (min)	HPLC Result (%)	*m*/*z*	Formula
**7**	5.2	17.03	428.1	C_22_H_23_FN_3_O_5_^+^
**8**	16.3	36.38	410.2	C_22_H_21_FN_3_O_4_^+^
**9**	16.7	19.56	412.2	C_22_H_23_FN_3_O_4_^+^

Notes: The numbering of the compounds is continuous and unique throughout this paper. Compound **9** (HPLC purity 99.64%) was subjected to the Fenton reaction-like condition at 60 °C for 4 h. The *m*/*z* values and formulae displayed are the protonated ions of the corresponding compounds.

**Table 5:** Product distribution of compound **12** subjected to 10% H_2_O_2_ solution spiked with 20 mM FeCl_3_ at 60 °C for 4 h.

Compound No. ^1^	Retention Time (min)	HPLC Result (%)	*m*/*z*	Formula
**10**	13.0	38.51	718.3	C_40_H_40_F_4_N_3_O_5_^+^
**11**	15.8	5.81	702.3	C_40_H_40_F_4_N_3_O_4_^+^
**12** ^2^	15.8	N.D. ^3^	704.3	C_40_H_42_F_4_N_3_O_4_^+^

Notes: ^1^. The numbering of the compounds is continuous and unique throughout this paper. ^2^. Compound **12** (HPLC purity 81.95%) was subjected to the Fenton reaction-like condition at 60 °C for 4 h. ^3^. N.D. = Not detected (LOD = 0.01%). The retention times of compounds **11** and **12** were the same and the identity of the peak was established as compound **11** by mass spectrometric analysis. The *m*/*z* values and formulae displayed are the protonated ions of the corresponding compounds.

## Data Availability

The original contributions presented in this study are included in the article/Supplementary Material. Further inquiries can be directed to the corresponding authors.

## Supplementary Materials

The following supporting information can be downloaded at https://www.mdpi.com/article/10.3390/molecules31030462/s1. The 1D 1H and 13C NMR spectra and 2D NMR of BAI and IDI, as well as the optimal production conditions for these compounds, are available in the “Supporting Materials”. Figure S1: Effect of different conditions on the formation of the benzaldehyde impurity (BAI) and indole impurity (IDI) from the key intermediate of silodosin (KIS): (A) reaction temperature, (B) reaction time, (C) H_2_O_2_ concentration, and (D) in the presence of FeCl_3_. Figure S2. Under the condition of 10% FeCl_3_ at 60 °C for 4 h, BAI was formed in an HPLC chromatographic yield of 2.3%, IDI was produced in an HPLC chromatographic yield of 12%. Figure S3: Under the optimal condition of 10% H_2_O_2_ solution at 60 °C for 4 h, ABI was produced in an HPLC chromatographic yield of 47.9%. Figure S4: Under the optimal condition of 10% H_2_O_2_ solution spiked with 20 mM FeCl_3_ at 60 °C for 4 h, IDI was formed in an HPLC chromatographic yield of 42.8%. Figure S5: The structures of KIS, BAI and IDI and numbering of the skeletons of the three compounds. Figure S6: gHSQC spectrum of BAI. C-10 (188.9 ppm) exhibited a correlation with H-10 (9.5 ppm). Figure S7: gHMBC spectrum of IDI. C-11 (205.9 ppm) correlated with H-10 (3.80 ppm) and H-12 (2.22 ppm).
