# Supplementary material for: Preparation of Two Process-Related Impurities of a Key Intermediate of Silodosin Under Baeyer–Villiger and Fenton Conditions
Source: Molecules. 2026 Jan 28;31(3):462. doi: 10.3390/molecules31030462 (PMC12898569; doi:10.3390/molecules31030462)
Supplement: Supplementary file 1 [file molecules-31-00462-s001.zip › molecules-4049865-supplementary.pdf]

Preparation of Two Process-Related Impurities of a Key Intermediate of Silodosin Under Baeyer–Villiger and Fenton Conditions

Wenbin Chen [1,*], Qiang Zhou [2], Junjun Zhang [1], Jianyang Jin [2], Juan Zhang [1], Jiangbo Xi [1], Zhengwu Bai [1,*] and Min Li [2,3,*]

[1] School of Chemistry and Environmental Engineering, Wuhan Institute of Technology, Wuhan 430073, China; 20081703@wit.edu.cn (J.Z.); zhang_juan@wit.edu.cn (J.Z.); jbxi@wit.edu.cn (J.X.)
[2] Center of Excellence for Modern Analytical Technologies (CEMAT), Zhejiang Huahai Pharmaceutical Co., Ltd., Linhai 317204, China; zhouqiang@huahaipharm.com (Q.Z.); jinjianyang@huahaipharm.com (J.J.)
[3] Huahai US, Inc., 700 Atrium Drive, Somerset, NJ 08873, USA
* Correspondence: chenwenbin1981@126.com (W.C.); zwbai@wit.edu.cn (Z.B.); minli88@yahoo.com (M.L.)

Supporting Materials

Formation of the benzaldehyde impurity (BAI) and indole impurity (IDI) in 10% H_2O_2 solutions under different temperature

The effects of temperature on the formation of BAI and IDI were investigated with 10% H_2O_2 concentration for 4 h. Hence, four parallel mixture were prepared according to the following procedure: 1 mL of KIS solution (the concentration of 3 mg/mL in acetonitrile/H_2O, 80/20, v/v) was mixed with 1 mL 30% H_2O_2 solution and 1 mL of sample diluent (acetonitrile/H_2O, 80/20, v/v). Each of the prepared solutions was subjected to temperatures at 40, 50, 60, and 70℃ for 4 h, respectively. As detailed in Fig. S1.A, the results show that the content of BAI increased from 1.6% to 47.9% within the temperature range of 40 to 60℃, while the content of IDI increased from 4.5% to 12.1%.

Consequently, the optimal synthesis conditions for BAI were determined to be 60°C, a 10% H_2O_2 concentration, and a reaction duration of 4 hours.

Formation of BAI and IDI in 10% H_2O_2 solutions at 60°C with different reaction times

Under a 10% H_2O_2 concentration and a reaction temperature of 60°C, the effect of the reaction time on the formation of BAI and IDI was investigated. As shown in **Fig. S1.B**, at 4 h of reaction time, the content of BAI initially increased when the reaction time increased from 3 h to 4 h. However, as the reaction time was further increased, both BAI and IDI did not exhibit a significant increase, instead, they showed a slight downward trend.

Impact of H_2O_2 concentration on the formation of BAI and IDI

The effect of less concentrated H_2O_2 solutions on BAI and IDI impurity formation was studied at 60°C for 4 hours. As shown in **Fig. S1.C**, BAI increased sharply from 1.7% to 7.4%, 27.5% and 47.9%, as the H_2O_2 concentration rose from 0.33%, 1%, 3.3% and 10%, respectively. In contrast, IDI showed a modest increase from 3.7% to 12.1%.

Impact of Varying $FeCl_3$ concentration on the formation of BAI and IDI

The effects of iron ion concentration on the formation of BAI and IDI were investigated under fixed conditions of 10% H_2O_2, 60°C, and a 4-hour reaction time (**Fig. S1.D**). The results demonstrate that the generation of BAI is predominantly dependent on the concentration of H_2O_2. Specifically, BAI was not observed as a major degradation product in the absence of H_2O_2 or at low iron ion concentrations. In contrast, the combined use of 20 mM $FeCl_3$ and 10% H_2O_2

resulted in an IDI content of 42.8%. When only a single oxidant was present, the IDI content was ~10%. When $FeCl_3$ was used as the sole oxidant, BAI yield reached only 2.3%, underscoring the limited contribution of iron-mediated oxidation in the absence of H_2O_2 (**Fig. S2**). This comparison indicates that the synergistic action of $FeCl_3$ and H_2O_2 significantly promotes the formation of IDI.

The HPLC chromatograms of BAI and IDI obtained under the optimized reaction conditions are presented in **Fig. S3** and **Fig. S4**, respectively.

NMR results of BAI

In the carbon spectrum of BAI, a total of 20 carbon atoms are identified, indicating a reduction of two carbon atoms compared to KIS (refer to **Fig.S5** for the structures of the relevant compounds).

In the gHSQC spectrum (**Fig. S6**), C-25 (188.9 ppm) exhibited a correlation with H-25 (9.55 ppm), where H-25 appeared as a singlet in the ^{1}H-NMR spectrum, indicating the presence of a single hydrogen atom. The chemical shift of H-25 at 9.55 ppm suggests that this hydrogen atom is highly active, which is consistent with the characteristic chemical shifts observed for aldehyde groups.

Furthermore, H-17 (3.01 ppm) and H-15 (3.81 ppm) each exhibited signals corresponding to two hydrogen atoms, closely resembling those of the corresponding positions in KIS. This observation supports the conclusion that dehydrogenation did not occur on the dihydroindole ring, thereby confirming the structure of BAI.

High resolution MS: 353.1324 [M+H$^+$]; theoretical for $C_{20}H_{18}FN_2O_3^+$, calcd. 353.1296 [M+H$^+$], error of 8 ppm.

^{1}H NMR (DMSO-*d6*, 400 MHz): δ 2.12 (s, 2H, CH_2), 3.01 (t, 2H, J=8.61, CH_2), 3.81 (m, 2H, CH_2), 3.84 (m, 2H, CH_2), 4.38 (t, 2H, J=5.87, CH_2), 7.29 (m, 2H, Ar-H), 7.46 (s, 1H, Ar-H), 7.79 (s, 1H, Ar-H), 8.00 (m, 2H, Ar-H), 9.55 (s, 1H, CH).

^{13}C NMR (DMSO-*d6*, 100 MHz): δ 188.9, 166.5, 165.1, 164.0, 155.8, 140.2, 134.4, 132.3, 132.2, 126.3, 126.3, 126.2, 125.0, 118.4, 116.0, 115.7, 85.2, 63.0, 52.9, 44.1, 26.4, 25.7.

2D NMR results of IDI

The ^{1}H and ^{13}C NMR data revealed that C-26 (206.1 ppm) correlated with H-25 (3.87 ppm) and H-28 (2.28 ppm) in the gHMBC spectrum (**Fig. S7**). Notably, the chemical shifts of H-28 were consistent with the presence of three hydrogen atoms. The chemical shifts of C-26, H-25, and H-28 closely resembled those observed at corresponding positions in KIS, thereby confirming the presence of the ketone moiety.

Furthermore, C-17 (102.0 ppm) exhibited a correlation with H-17 (6.58 ppm) as depicted in the gHSQC spectrum, where H-17 was characterized by a single hydrogen atom. Similarly, C-15 (132.1 ppm) correlated with H-15 (7.57 ppm), which also represented a single hydrogen atom. Both protons are situated downfield primarily due to double bond formation resulting from dehydrogenation within the dihydroindole ring. These findings support the structural characterization of IDI.

High resolution MS: 379.1458 [M+H$^+$]; theoretical for $C_{22}H_{20}FN_2O_3^+$, calcd. 379.1452 [M+H$^+$], error of 2 ppm.

^{1}H NMR (DMSO-*d6*, 400 MHz): δ 2.15 (s, 3H, CH_3), 2.28 (m, 2H, CH_2), 3.87 (s, 2H, CH_2), 4.29 (t, 2H, J=5.87, CH_2), 4.64 (t, 2H, J=6.85, CH_2), 6.58 (d, 1H, J=3.13, CH), 7.28 (m, 2H, Ar-H), 7.45 (d, 1H, J=1.17, Ar-H), 7.57 (d, 1H, J=3.13, Ar-H), 7.70 (d, 1H, J=1.57, Ar-H), 7.91 (m, 2H, Ar-H).

^{13}C NMR (DMSO-*d6*, 100 MHz): δ 206.1, 166.3, 164.7, 163.8, 132.2, 132.2, 132.1, 130.3, 129.6, 127.6, 126.1, 126.1, 126.0, 118.3, 115.7, 115.5, 102.0, 92.3, 62.4, 48.4, 44.1, 29.8, 29.5.

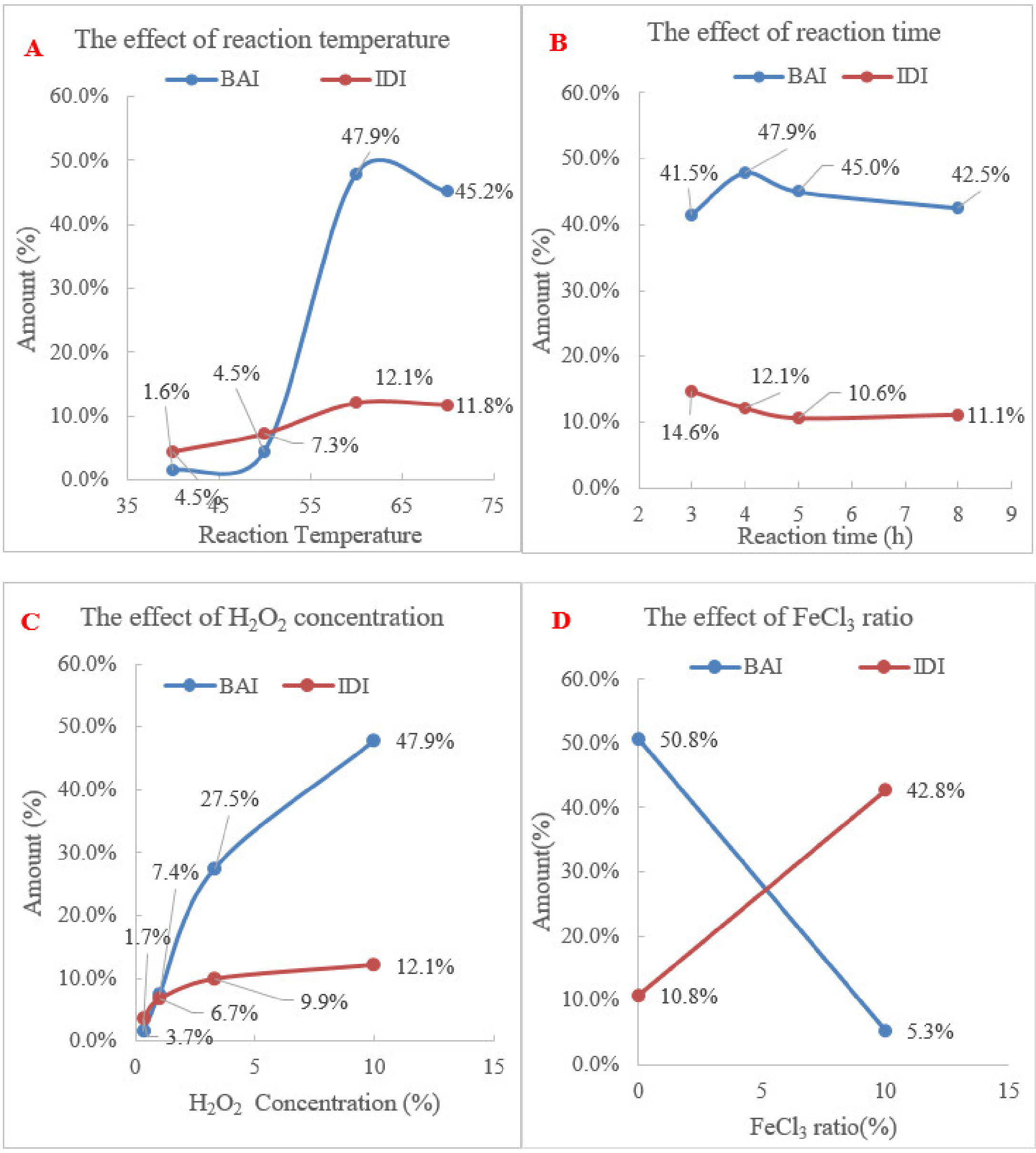

Figure S1. Effect of different conditions on the formation of the benzaldehyde impurity (BAI) and indole impurity (IDI) from the key intermediate of silodosin (KIS): (A) reaction temperature, (B) reaction time, (C) H_2O_2 concentration, and (D) in the presence of $FeCl_3$.

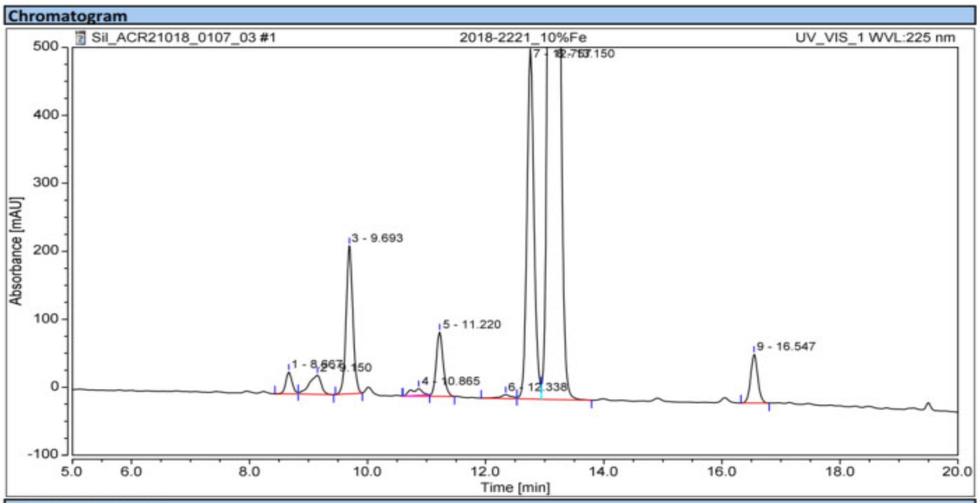

Integration Results

No.	Peak Name	Retention Time min	Area mAU*min	Height mAU	Relative Area %	Relative Height %	Amount n.a.
1		8.667	4.083	32.349	0.69	0.94	n.a.
2		9.150	6.612	28.431	1.12	0.83	n.a.
3		9.693	28.529	217.633	4.84	6.32	n.a.
4		10.865	2.206	9.711	0.37	0.28	n.a.
5		11.220	13.430	94.601	2.28	2.75	n.a.
6		12.338	1.289	5.773	0.22	0.17	n.a.
7		12.757	71.075	514.921	12.06	14.96	n.a.
8		13.150	451.966	2466.170	76.71	71.66	n.a.
9		16.547	10.009	71.876	1.70	2.09	n.a.
**Total:**			**589.198**	**3441.464**	**100.00**	**100.00**	

Figure S2. Under the condition of 10% $FeCl_3$ at 60°C for 4 hours, BAI was formed in an HPLC chromatographic yield of 2.3%, IDI was produced in an HPLC chromatographic yield of 12%.

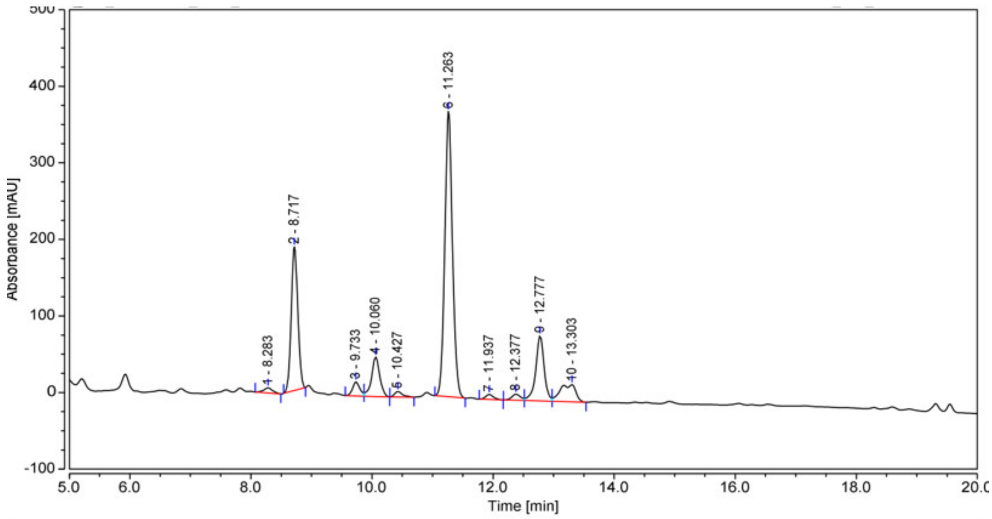

Integration Results

No.	Peak Name	Retention Time min	Area mAU*min	Height mAU	Relative Area %	Resolution (USP)	Amount n.a.
1		8.283	1.117	6.702	0.98	n.a.	n.a.
2		8.717	23.620	187.518	20.76	1.90	n.a.
3		9.733	2.535	18.379	2.23	4.88	n.a.
4		10.060	8.096	51.802	7.12	1.45	n.a.
5		10.427	1.120	7.206	0.98	1.59	n.a.
6		11.263	54.510	372.630	47.92	3.67	n.a.
7		11.937	0.977	6.418	0.86	2.89	n.a.
8		12.377	1.308	8.030	1.15	1.75	n.a.
9		12.777	13.731	85.084	12.07	1.59	n.a.
10		13.303	6.739	22.534	5.92	1.62	n.a.
**Total:**			**113.7535**	**766.3042**	**100.00**	**21.34**	

Figure S3. Under the optimal condition of 10% H_2O_2 solution at 60°C for 4 hours, ABI was produced in an HPLC chromatographic yield of 47.9%.

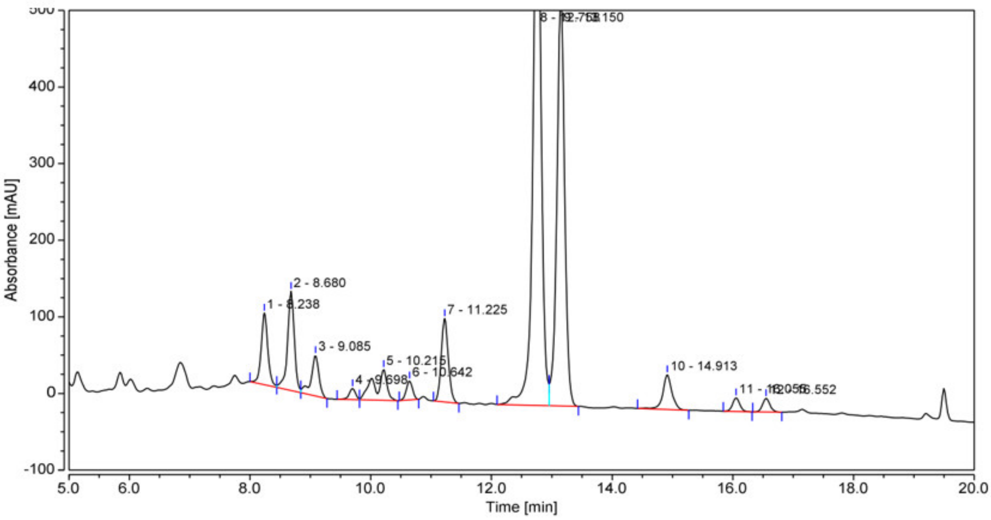

Integration Results							
No.	Peak Name	Retention Time min	Area mAU*min	Height mAU	Relative Area %	Relative Height %	Amount n.a.
1		8.238	11.966	93.559	4.29	5.03	n.a.
2		8.680	17.006	129.758	6.10	6.97	n.a.
3		9.085	7.998	53.186	2.87	2.86	n.a.
4		9.698	1.786	14.610	0.64	0.79	n.a.
5		10.215	9.530	40.605	3.42	2.18	n.a.
6		10.642	3.000	24.916	1.08	1.34	n.a.
7		11.225	14.728	108.851	5.28	5.85	n.a.
8		12.758	119.342	775.187	42.82	41.66	n.a.
9		13.150	80.408	538.867	28.85	28.96	n.a.
10		14.913	7.928	45.543	2.84	2.45	n.a.
11		16.055	2.446	17.855	0.88	0.96	n.a.
12		16.552	2.551	17.876	0.92	0.96	n.a.
Total:			278.691	1860.814	100.00	100.00	

Figure S4. Under the optimal condition of 10% H_2O_2 solution spiked with 20 mM $FeCl_3$ at 60°C for 4 hours, IDI was formed in an HPLC chromatographic yield of 42.8%.

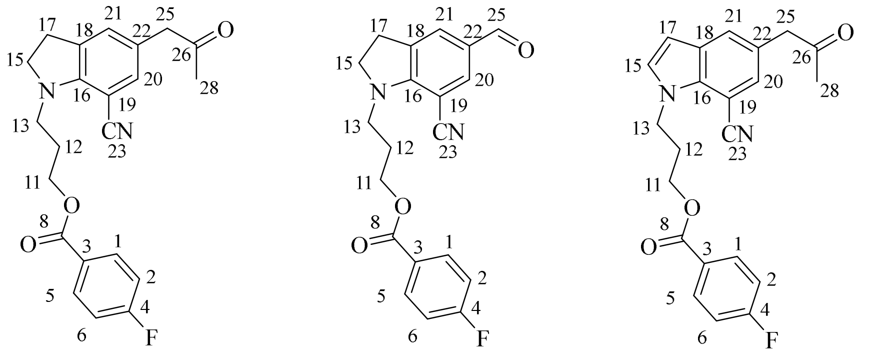

$C_{22}H_{21}FN_2O_3$
Exact Mass: 380.1536
Key Intermediate of Silodosin (KIS)

$C_{20}H_{17}FN_2O_3$
Exact Mass: 352.1223
Benzaldehyde Impurity (BAI)

$C_{22}H_{19}FN_2O_3$
Exact Mass: 378.1380
Indole Impurity (IDI)

Figure S5. The structures of KIS, BAI and IDI and numbering of the skeletons of the three compounds.

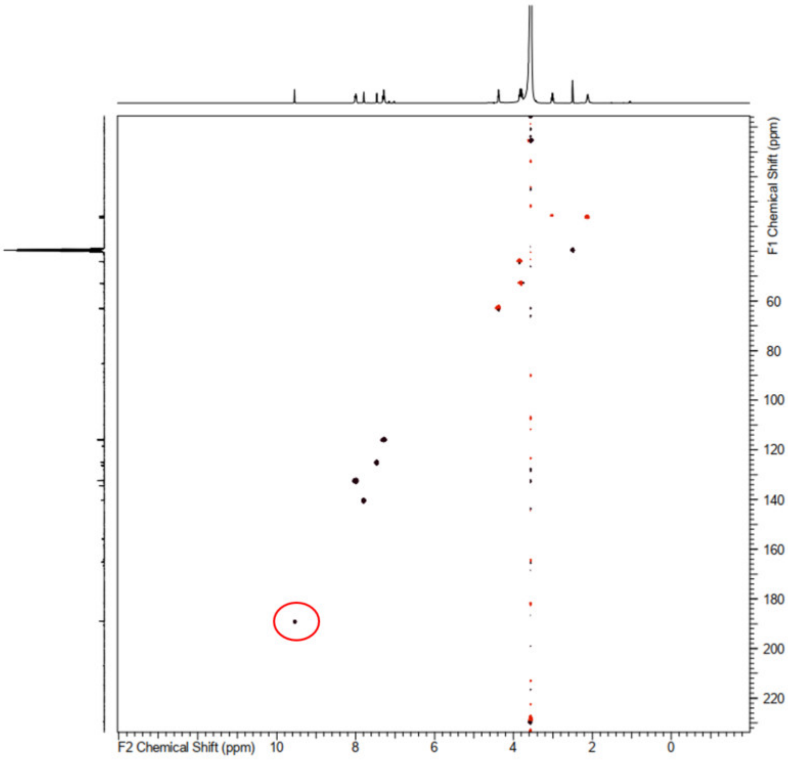

Figure S6. gHSQC spectrum of BAI. C-10 (188.9 ppm) exhibited a correlation with H-10 (9.5 ppm).

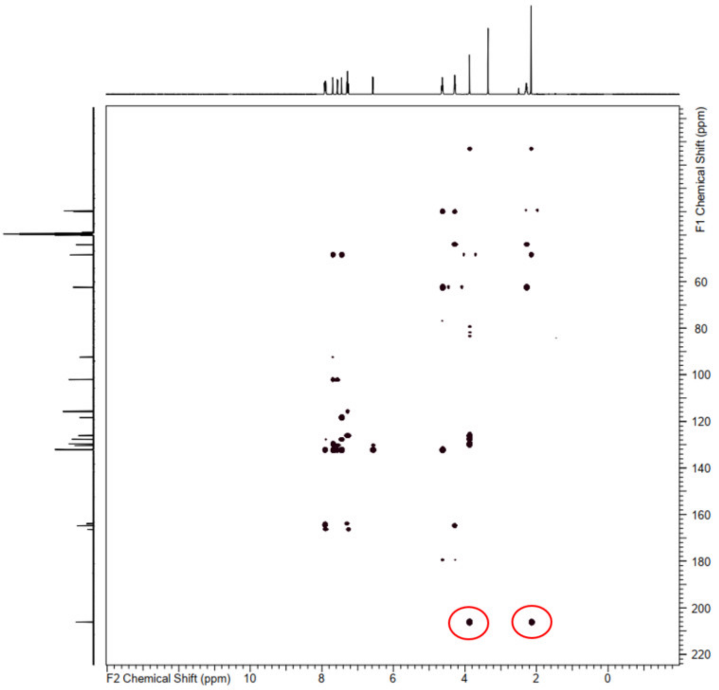

Figure S7. gHMBC spectrum of IDI. C-11 (205.9 ppm) correlated with H-10 (3.80 ppm) and H-12 (2.22 ppm).
